# Effective activation of antioxidant system by immune-relevant factors reversely correlates with apoptosis of *Eisenia andrei* coelomocytes

**DOI:** 10.1007/s00360-016-0973-5

**Published:** 2016-02-27

**Authors:** J. Homa, M. Stalmach, G. Wilczek, E. Kolaczkowska

**Affiliations:** Department of Evolutionary Immunology, Institute of Zoology, Jagiellonian University, Gronostajowa 9, 30-387 Kraków, Poland; Department of Animal Physiology and Ecotoxicology, Faculty of Biology and Environmental Protection, University of Silesia, Bankowa 9, 40-007 Katowice, Poland

**Keywords:** PMA, LPS, Zymosan, *Micrococus luteus*, Apoptosis, Antioxidative enzymes

## Abstract

Oxidative stress is harmful to the microbes but also to the host, and may result in bystander damage or death. Because of this, respiratory burst triggered in phagocytes by pathogens is counteracted by production of antioxidative factors. The aim of this work was to examine effectiveness of the latter system in earthworms *Eisenia andrei* by induction of reactive oxygen species, lipofuscin and phenoloxidase by natural (LPS, zymosan, *Micrococus luteus*) and synthetic (phorbol ester, PMA) stimulants. The compounds impaired numbers, viability (increased apoptosis) and composition of coelomocytes, and triggered the antioxidant activity of catalase and selenium-dependent glutathione peroxidase. The natural pathogenic compounds, unlike PMA, strongly activated antioxidative responses that diminished cell apoptosis. Moreover, repeated exposure to the same or different pathogenic compounds did not induce respiratory burst exhausted phenotype showing that coelomocytes are constantly at bay to withstand numerous infections. The current study reveals importance and efficiency of the oxidative–antioxidative systems in annelids but also confirms its evolutionary conservatism and complexity even in lower taxa of the animal kingdom.

**Electronic supplementary material:**

The online version of this article (doi:10.1007/s00360-016-0973-5) contains supplementary material, which is available to authorized users.

## Introduction

Reactive oxygen species (ROS) are formed by every cell in the body during respiration and other physiological processes, foremost, however, ROS are generated with a purpose to fight infectious pathogens during so called respiratory burst (Salganik 2001).

In recent years many studies have been carried out to determine the cellular and biochemical processes involved in the immune function of invertebrates (as reviewed in Söderhäll 2010). We know that they do not produce antibodies, lymphocytes or other characteristic elements of the adaptive immune response. However, despite this fact, they can defend themselves against pathogens as effectively as vertebrates due to a very well formed innate immunity (Cooper 1996; Bilej et al. 2000; Kauschke et al. 2007). Earthworm immune responses are almost entirely supported by circulating coelomocytes which are subdivided to different types of amoebocytes and eleocytes (Cooper 1996; Kurek et al. 2007), and humoral molecules (Bilej et al. 2000). Amoebocytes are involved in the immune response by phagocytosis, encapsulation and cytotoxicity (NK cell-like activity) of pathogens (Cooper 1996). And eleocytes synthesize and release immune humoral molecules such as agglutinins and opsonins which lead to immobilization of the pathogen (Cooper 1996; Bilej et al. 2001). Moreover, coelomic fluid of earthworms contains enzymes such as antimicrobial proteases that are also involved in the process of prophenoloxidase cascade (pro-PO) activation which requires ROS (Beschin et al. 1998; Kauschke et al. 2007). Active phenoloxidase (PO) is a key enzyme of melanization, catalyzing the oxidation of phenols to quinones which subsequently polymerize into melanin deposited in brown bodies (Valembois et al. 1994). In annelids, in response to a number of invading pathogens (protozoa, nematodes), coelomocytes form capsules in a process called encapsulation (Valembois et al. 1992). Namely, encapsulated material is surrounded by layers of coelomocytes, which form a capsule (a brown body) in which pigments such as melanin and lipofuscin are synthesized (Valembois et al. 1994). Subsequently, in a positive feedback loop, the pigments induce further production of ROS (Valembois and Lassègues 1995). Release of free radicals may also be initiated by accumulation of lipoproteins that form so-called “age pigment” described also as lipofuscin (Katz and Robinson Jr 2002; Terman and Brunk 2004). Lipofuscin is synthesized early on during formation of multicellular aggregates which finally turn to brown bodies, and at later stages melanin is also produced (Valembois et al. 1994). The synthesis of the pigments in the earthworms is the last stage in neutralization of pathogens and at this stage the pigments inactivate ROS.

The enhanced production of ROS, including superoxide and hydrogen peroxide, during environmental stress can pose a threat to cells by causing peroxidation of lipids, oxidation of proteins, damage to nucleic acids, activation of programmed cell death (apoptosis) pathway and ultimately leading to death of the cells (Green and Reed 1998). For this reason, cells have a variety of defense mechanisms to resist the harmful effects of ROS. They include superoxide dismutase (SOD) which catalyzes conversion of superoxide into hydrogen peroxide and oxygen, and glutathione peroxidases and catalases which then degrade hydrogen peroxide (Saint-Denis et al. 1998). However, environmental stress, including infection can cause either enhancement or depletion of the antioxidant activity (e.g. Sigfrid et al. 2003). Thus the antioxidative system does not have to be switched on or it can be poorly effective depending on the stimulant.

Up till now ROS production and antioxidative mechanisms of invertebrates have been investigated mostly in studies dedicated to immunotoxicology of pollutants (Velki and Hackenberger 2013). The objective of this study was to monitor the immune reaction of earthworms after induction of oxidative stress with numerous biologically relevant microbe-derived compounds with which the earthworms are in contact during their life span. Our main question being if the respiratory burst would always induce the counter anti-oxidative response and if the latter would effectively protect the host. We also sought to observe how repeated stimulation, occurring in natural environment, will affect earthworm capacity to cope with it in terms of oxidative burst. In fact invertebrate immune system is naturally activated by bacterial or fungal cell wall components (Vargas-Albores and Yepiz-Plascencia 2000). Here we concentrated on diverse immunostimulants including one of the most potent activators of the immune system, lipopolysaccharide (LPS) from *Escherichia coli*. Moreover, we tested Gram-positive *Micrococus luteus* present in soil and water, and β-glucan of yeast cell wall (zymosan). As a reference we used a synthetic stimulant, phorbol-12-myristate-13-acetate (PMA) which is one of strongest inducers of respiratory burst (e.g. Haugland et al. 2012).

Here we report on a correlation between triggering protective antioxidant mechanisms and improving survival at the cellular and organismal levels. Comparison of the current results with data from other invertebrates and vertebrates clearly shows universalism of this phenomenon.

## Materials and methods

### Animals and exposure condition

Adult (clitellate) earthworms (0.55–0.75 g body weight) of *Eienia andrei* (Sav.) were collected from the stockbreeding maintained in the Institute of Zoology of the Jagiellonian University, kept in controlled laboratory conditions (21 ± 1 °C; 12:12 LD) in a commercial soil (PPUH Biovita, Poland). On the experimental day, each earthworm was washed with water, and then injected with 20 µl of stimulant solution into the coelomic cavity (1 cm behind the clitellum). The stimulants were prepared in sodium chloride solution 0.9 % (Baxter Terpol, Poland) and the following compounds were used: phorbol 12-myristate 13-acetate-PMA (PMA, 0.1 µg/ml), lipopolysaccharide from *E. coli* 0111:B4 (LPS, 1 mg/ml), zymosan A from *Saccharomyces cerevisiae* (Z, 1 mg/ml) or *Micrococcus luteus* (Ml, 1 mg/ml). All stimulants were purchased from Sigma (St. Louis, Mo., USA). LPS was used, rather than live *E. coli*, as it is the strongest compound/stimulating agent of all Gram^−^ bacteria. After injection, earthworms were placed individually in 15 ml vials filled with filter paper that was soaked with water (Homa et al. 2013, modified). Subsequently, coelomocytes were collected 24 or 72 h later for analyses. Control (CTR) animals were injected by sodium chloride solution (0.9 % NaCl). In addition, some control animals were kept in a commercial soil.

### Harvesting and determination of the number and composition of coelomocytes

24 or 72 h after injection, the earthworms were stimulated for 1 min with a 4.5 V electric current to expel coelomic fluid with coelomocytes through the dorsal pores according to the procedure described previously (Homa et al. 2008). Cells were collected into 0.05 mM Sörensen buffer (Na_2_HPO_4_–KH_2_PO_4_, POCh, Gliwice, Poland), pH 7.4, as it is preferred for antioxidant studies (Pothi 2013; Wilczek et al. 2013). Proper controls were run simultaneously, i.e. some cells were collected into RPMI or normal earthworm Lumbricus Balanced Salt Solution (LBSS, Fugère et al. 1996) and then NBT test was performed. No differences were detected between either of the tested solutions. Obtained cells were counted with a haemocytometer and their composition was evaluated based on morphology of amoebocytes (A) and eleocytes (E) (Homa et al. 2007).

### Flow cytometry analysis of coelomocytes complexicity

To determine a cell composition of coelomocytes by flow cytometry, the coelomic fluid samples were analyzed with FACScalibur (BD Biosciences) equipped with CellQuest software (Becton–Dickinson, San Diego, CA). During analytical experiments, 10,000 thresholded events per earthworm sample were collected and analyzed on the basis of their forward scatter (FSC) (for cell size) and side scatter (SSC) (cell granularity) properties. Fluorescence FL1-H was recorded for estimation of autofluorescent (AF) eleocytes (E).

### Flow cytometric quantification of apoptosis with Anexin V and 7AAD dyes

Apoptotic coelomocytes collected from the in vivo experiments (as in “Number, composition and viability of coelomocytes in response to immunostimulants”) were quantitated with the Annexin V-PE Apoptosis Detection Kit I (BD Pharmingen) that enables cell staining with Annexin V (AnxV, binds to phosphatidylserine exposed on the outer membrane of apoptotic cells) and 7-aminoactinomycin (7AAD) (which enters all dead cells) (Zimmermann and Meyer 2011; Pragya et al. 2014). The cells (1 × 10^6^/ml) were resuspended in binding buffer provided by the manufacturer. Then 5 µl of Annexin V-PE and 5 µl of 7AAD were added and incubated for 15 min at room temperature in the dark. Measurements were performed with a FACScalibur flow cytometer (BD Biosciences) equipped with CellQuest software (Becton–Dickinson). Annexin V-PE was measured in FL-2, and 7AAD in the FL-3 channel. Negative control cells, labelled with Annexin V-PE only (no 7AAD) and negative control cells, labelled with 7AAD only (no Annexin V-PE) were used for compensation. AnxV^+^/7AAD^−^ cells were considered early apoptotic and AnxV^+^/7AAD^+^ late apoptotic. Viable cells were double negative and necrotic cells were only positive for 7AAD^+^.

### Flow cytometric analysis of mitochondrial depolarization by MitoPT^®^ TMRE assay

The TMRE mitochondrial membrane potential kit applies TMRE (tetramethylrhodamine, ethyl ester) to label active mitochondria. TMRE is a cell permeant, positively-charged, red–orange dye that readily accumulates in active mitochondria due to their relative negative charge. Depolarized or inactive mitochondria have decreased membrane potential and fail to sequester TMRE (Jandova et al. 2013).

Coelomocytes (1 × 10^6^/ml) directly collected from the earthworms were incubated with MitoPT^®^ TMRE (100 nMol/1 ml cell samples, ImmunoChemistry Technologies, USA) in RT for 30 min (protected from light). After incubation the cells were centrifuged for a wash step (300×*g*, 5 min) and finally resuspended in 0.5 ml assay buffer (provided by the manufacturer), and then used for measurement of the TMRE staining of mitochondria.

The measurement of polarization of mitochondrial membrane was performed with a FACSCalibur flow cytometer. During analytical experiments, 10,000 thresholded events per earthworm sample were collected and analyzed on the basis of TMRE fluorescence FL2-H (orange fluorescence). All Flow cytometry data were analyzed using WinMDI 2.9 software (Joe Trotter, http://facs.scripps.edu).

### Respiratory burst

The intensity of respiratory burst in coelomocytes was measured with the nitroblue tetrazolium (NBT) as described previously (Chadzinska et al. 2009). Superoxide reduces nitroblue tetrazolium to a blue insoluble product known as formazan, which is detected in intracellular deposits (Kettle and Winterbourn 1997). Suspension of coelomocytes (1 × 10^6^/ml) was incubated with NBT (10 mg/ml, Sigma-Aldrich) for 1 h, and then the reaction was stopped with methanol. The plates were air-dried, and 120 μl of 2 N potassium hydroxide and 140 μl of dimethyl sulphoxide (DMSO) were added to each well to extract the dye. Next, the optical density (OD) was recorded with an ELISA Reader (micro ELISA Reader Expert Plus, ASYS Hitach GmbH, Austria).

### Activity of antioxidant enzymes: catalase (CAT) and peroxidises (GPOX and GSTP)

Samples of coelomic fluid containing coelomocytes, prepared in 0.05 M Sörensen buffer were stored at −20 °C prior to enzymatic assays (Wilczek et al. 2013), and after thawing the cells were centrifuged (10,000 rpm, 10 min 4 °C) to release the enzymes from the cells. Samples were collected from earthworms treated as described in “Number, composition and viability of coelomocytes in response to immunostimulants”.

#### Catalase

Catalase (CAT; EC 1.11.1.6) activity was determined as a degradation of H_2_O_2_ by the enzyme. In particular, it was measured as a decrease in absorbance at 25 °C (thermal coefficient Q10 = 1.1) for 30 s at 230 nm (*e* = 71.0 ODUM_1_cm_1) in 50 μl 0.05 mM Sörensen buffer, pH 7.4, containing 10 mM H_2_O_2_ (approx. 30 %, Sigma-Aldrich). The reaction was initiated by adding 100 µl of a sample. On figures, the enzymatic activity is expressed in micromoles of hydrogen peroxide reduced per minute per milligram of protein (Wilczek et al. 2013). Protein content was measured according to Bradford (1976) using bovine albumin (protein content >95 %, Fluka) as a standard.

#### Glutathione peroxidases

Both selenium-dependent (GPOX) and selenium-independent glutathione peroxidases (GSTPx) (EC 1.11.1.9) were determined spectrophotometrically at 340 nm, measuring the rates of reduction of either (1) hydrogen peroxide (H_2_O_2_, approx. 30 %, Sigma-Aldrich) for GPOX, or (2) cumine oxide (cumOOH, C_9_H_12_O_2_, approx. 80 %, Sigma Aldrich) for GSTPX in the presence of NADPH (min 93 %, Sigma Aldrich) and glutathione reductase (GR, 93 U/mg protein, Fluka BioChemica). Briefly, 10 µl of coelomic fluid containing coelomocytes was added to mixture containing 0.05 M Sörensen buffer, 2 mM EDTA, 2 mM sodium azide, 10 mM reduced glutathione (GSH), 1 IU of glutathione reductase, 2.5 mM NADPH, to reach a final volume of 100 µl. The reaction was started with addition of either 2.5 mM H_2_O_2_ or 15 mM cumOOH. Results are expressed in nmol NADPH/min/mg protein (according to Wilczek et al. 2013).

### Estimation of phenoloxidase activity (PO) in coelomocytes

The PO activity was estimated as production of dopachrome, product of the oxidation of l-DOPA (l-3,4-dihydroxyphenylalanine) by PO (e.g. Procházková et al. 2006). Coelomic fluid containing coelomocytes was frozen (−20 °C) and after thawing centrifuged (10,000 rpm, 10 min 4 °C) to release the enzyme from the cells. PO activity was estimated according to Procházková et al. (2006) with some modifications. Briefly, 10 μl of the samples was mixed with 90 μl of 0.1 M-Tris/HCl buffer (pH = 8). Then 10 μl of l-DOPA (3 mg/ml in distilled water, Sigma-Aldrich) was added (all steps were performed on ice). Absorbance was measured immediately after adding l-DOPA, and again in 60 min intervals for 6 h, and after 24 h incubation in RT (protected from light). Data for 24 h are shown in figures. The optical density (OD) was recorded with the ELISA reader at 490 nm.

### Detection of lipofuscin in coelomocytes

Lipofuscin content was detected using the modified Schmorl reaction (Moore 1988). Suspension of coelomocytes was incubated for 1 h to let the cells adhere to a 96-well flat-bottomed plate (1 × 10^6^/ml, 100 µl of cells per well). Next, the cells were fixed for 15 min in calcium-formalin. After that the plates were rinsed in distilled water and stained with the reaction medium. The latter contained ferric chloride (1 % aqueous solution) and potassium ferricyanide (1 % aqueous solution) in a ratio of 3:1 (freshly prepared, POCh). The coelomocytes were stained for 5 min in the solution, then rinsed for 1 min in 1 % acetic acid, followed by distilled water. The blue reaction product, indicating lipofuscin, was monitored microscopically (data not showed). The extraction of the dye was performed with 1 M NaOH and the OD was obtained with the ELISA reader at 490 nm.

### Data analysis and statistics

Results are expressed as means ± standard errors (*X* + SE). On figures, data are recalculated as percentage of control (100 %). Significant differences between means were evaluated using one-way ANOVA test. The level of significance was established at *p* < 0.05. Statistical comparisons of the same groups at different time points were performed by Student’s *t* test, at *p < 0.05; **p < 0.01; ***p < 0.001.

## Results

### Number, composition and viability of coelomocytes in response to immunostimulants

**Fig. 1:**
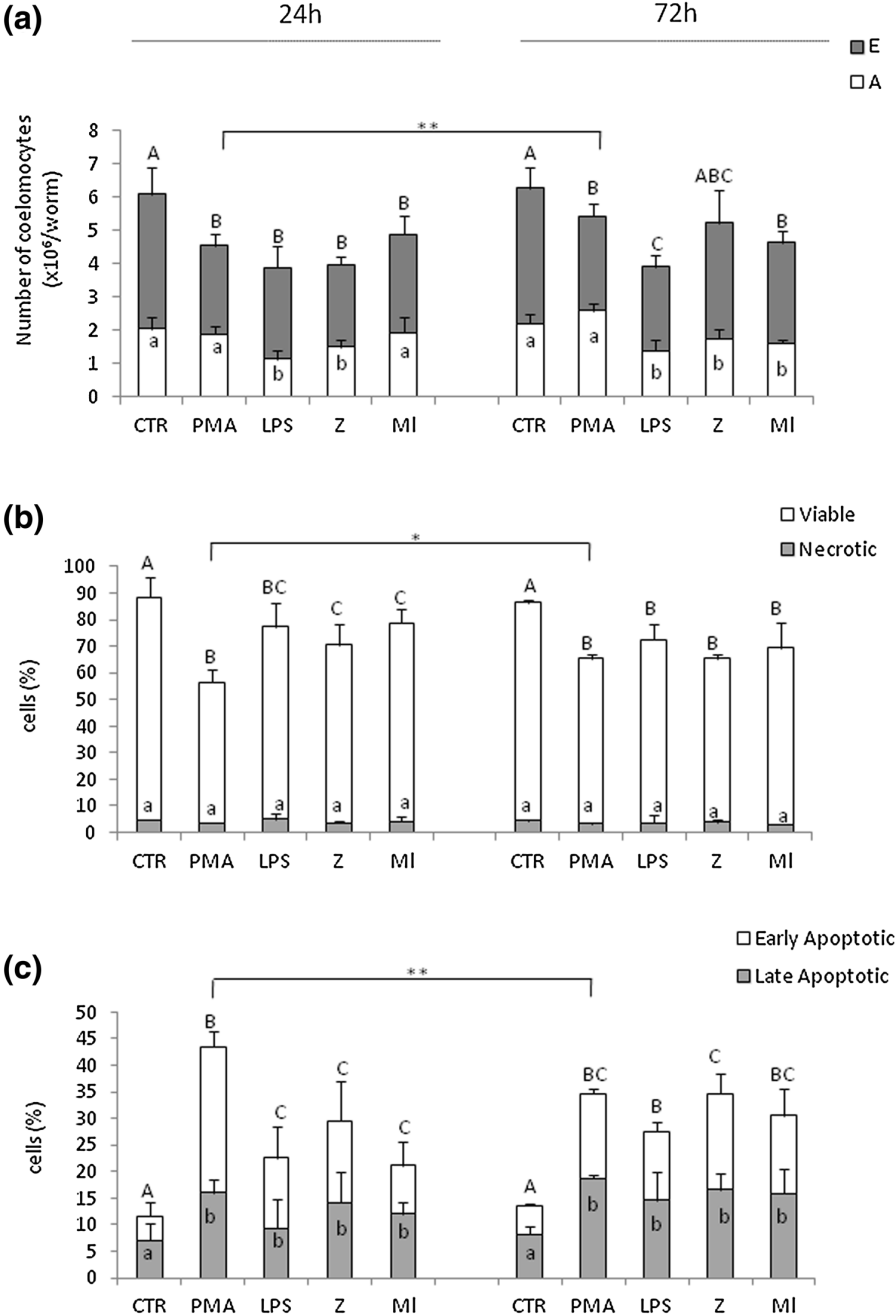
Numbers of coelomocytes, eleocytes (E) and amoebocytes (A) (**a**), and their viability (**b**, **c**). Coelomocytes were retrieved from earthworms *E. andrei* after 24 or 72 h since injection with sodium chloride (control—CTR) or PMA (0.1 µg/ml), LPS (1 mg/ml), zymosan (Z, 1 mg/ml) or *M. luteus* (Ml, 1 mg/ml). Coelomocyte viability was assessed by flow cytometric analysis of cells stained with annexin V (AnxV) and 7AAD: **b** viable (AnxV^−^/7AAD^−^) and necrotic (AnxV^−^/7AAD^+^) cells; **c** early apoptotic (AnxV^+^/7AAD^−^) and late apoptotic (AnxV^+^/7AAD^+^) cells. Mean + SE, *n* = 12–16 (3–5 earthworms per experiment, each experiment was repeated 3–4 times). Different letters (e.g. **a** vs. **b** or *A* vs. *B*) indicate statistically significant differences between the groups at *p* < 0.05, according to one-way ANOVA, *differences statistically significant between the same groups at different time points, at *p < 0.05; **p < 0.01, according to *t* test

Coelomocytes are the first line of cellular response of earthworm immune system and their number, type and activity can vary during immune responses (Homa et al. 2013; Irizar et al. 2015). In control animals (CTR) number and composition of coelomocytes were comparable at 24 and 72 h, but all of used stimulants (PMA, LPS, zymosan—Z, *M. luteus*—Ml) had statistically significantly decreased coelomocyte numbers in comparison to the control animals at either time point (Fig. 1a). Moreover, composition of coelomocytes, both amoebocytes (A) and eleocytes (E), was changed. While this effect was detected at both time points, and after all of used immunostimulants, the effect was not uniformed. In particular, amoebocytes were most affected by the LPS treatment as their numbers decreased by 45 and 37 % at 24 and 72 h, respectively. In contrast, PMA did not reduce the number of amoebocytes, and even statistically significantly increased their numbers (by about 17 %) at 72 h, in comparison to control (Fig. 1a). In contrast, in the case of eleocytes, PMA was the most toxic immunostimulant. It reduced the number of cells by approximately 35 and 31 % at 24 and 72 h, respectively. A similar effect was also observed after zymosan treatment (Fig. 1a).

It has been demonstrated that coelomocytes obtained from earthworms treated with immunostimulants are less vital (Homa et al. 2013). Our flow cytometric analyses showed that app. 90 % of coelomocytes were viable in the control (CTR) group, as detected upon staining with Annexin V (AnxV) and 7AAD (Suppl. Fig. 1 showing exemplary dot plots, and Fig. 1b). However, application of all stimulants significantly decreased the viability. The stimulants did not induce necrosis, and instead promoted apoptosis (Fig. 1c). We distinguished two stages of this reaction, early apoptosis (AnxV^+^/7AAD^−^) and late apoptosis (AnxV^+^/7AAD^+^), and detected that PMA in particular induced numerous coelomocytes to enter the apoptotic pathway after 24 h of stimulation (Fig. 1c). While similar numbers of eleocytes and amoebocytes were undergoing apoptosis upon LPS, Z and Ml stimulation, more amoebocytes than eleocytes were apoptotic in response to PMA (Table 1).

**Table 1:** Summary of effects induced by immunostimulation on tested cells, amoebocytes and eleocytes, and their oxidative and antioxidative potential

	PMA		LPS		Z		Ml	
	24 h	72 h	24 h	72 h	24 h	72 h	24 h	72 h
Amoebocytes	−	↑	↓↓	↓↓	↓	↓	↓	↓
Eleocytes	↓	↓	↓	↓	↓	↓	↓	↓
Apoptosis	↑↑A, ↑E	↑↑A, ↑E	↑A, ↑E	↑A, ↑E	↑A, ↑E	↑A, ↑ E	↑A, ↑E	↑A, ↑E
Δψm	↑↑A, ↑E	↑↑A, −E	↑A, ↑E	↑A, ↑E	↑A, −E	↑↑A, ↑ E	↑A, −E	↑A, −E
ROS	↑↑	↑	↑↑	↑	↑↑	↑	↑↑	↑
CAT	↑	−	↑↑	−	↑	−	↑↑	−
GPOX	−	−	↑↑	−	↑	−↑	↑↑	−
GSTPX	−	−	−	−	−	−	−	−
Proliferation	nt	↑↑A	nt	↑A	nt	nt	nt	nt

Proliferation, results from (Homa et al. 2013)

PMA, phorbol 12-myristate 13-acetate; LPS, lipopolysaccharide from *E. coli* 0111:B4; Z, zymosan A from *S. cerevisiae*; Ml, *Micrococcus luteus*; Amoebocytes, A; Eleocytes, E; apoptosis, cells stained with Annexin V; Δψm, mitochondrial membrane depolarization; ROS, respiratory burst measured with NBT test; CAT, catalase; GPOX, selenium-dependent glutathione peroxidases; selenium-independent glutathione peroxidases, GSTPX; nt, not tested; (**↑↓**), effect; (**−**), no effect

### Mitochondrial membrane potential depolarization in coelomocytes in response to immunostimulants

**Fig. 2:**
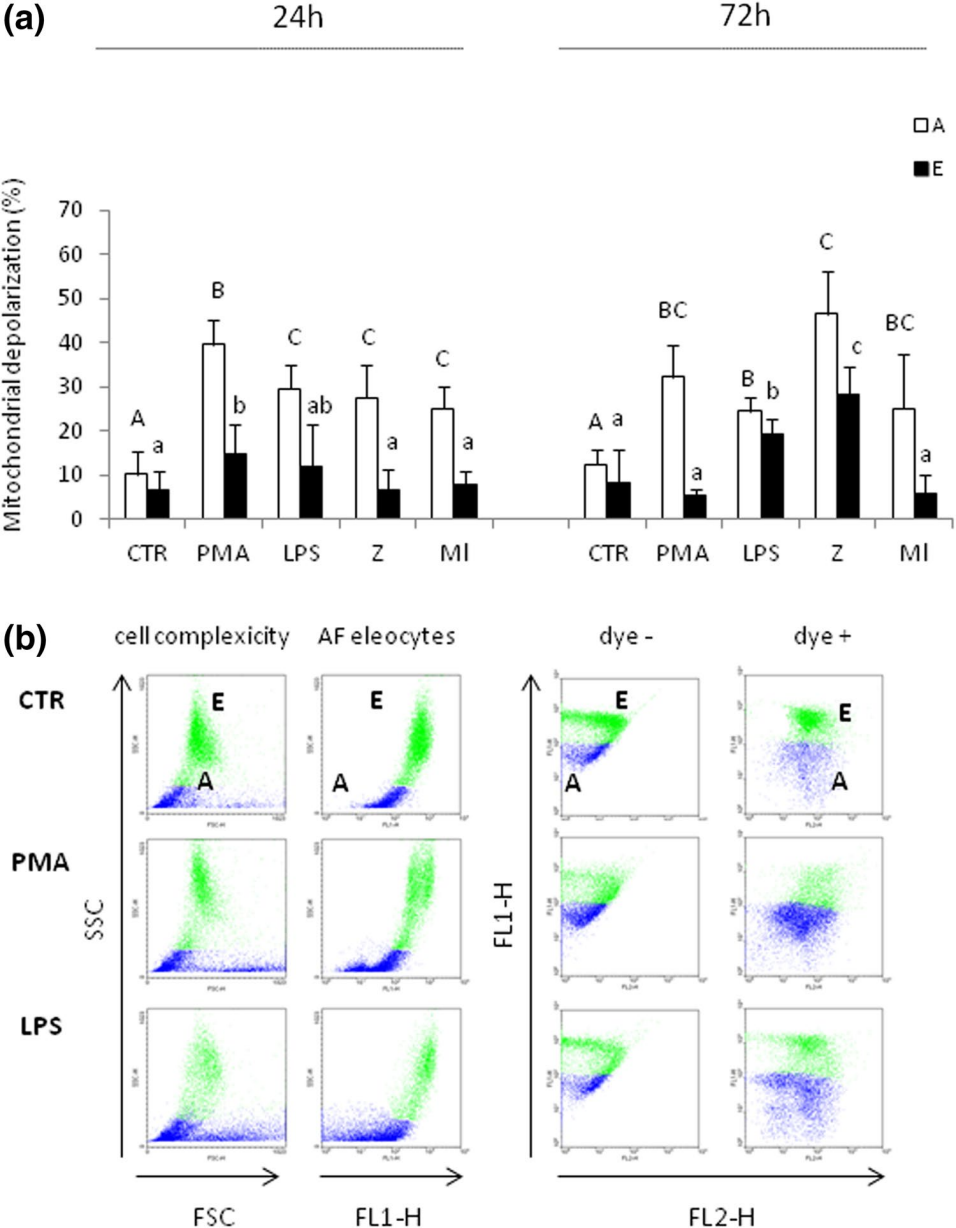
Flow cytometric analysis of mitochondrial membrane potential depolarization (MiToPT^®^ TMRE assay) in coelomocytes. The cells were retrieved from earthworms *E. andrei* after 24 or 72 h since injection with sodium chloride (control—CTR) or PMA (0.1 µg/ml), LPS (1 mg/ml), zymosan (Z, 1 mg/ml) or *M. luteus* (Ml, 1 mg/ml). Subsequently the mitochondrial membrane polarization was tested ex vivo and it is expressed on graphs as percentage of its depolarization against the control cells (**a**). Mean + SE, *n* = 5–7 (2–3 earthworms per experiment, each experiment was repeated 3–4 times). Different letters (e.g. **a** vs. **b** or *A* vs. *B*) indicate statistically significant differences between the groups at *p* < 0.05, according to one-way ANOVA; **b** representative density plots of coelomocytes retrieved from a control worm (CTR) and animals stimulated with PMA and LPS for 24 h in which the mitochondrial potential was tested; amoebocytes (A) and eleocytes (E). *Dot-plots* (*left* to *right*) show: cell complexity (cell granularity SSC-H vs. cell size FSC-H) and autofluorescence of eleocytes (AF) (cells granularity SSC-H vs. FL1-H). *Dotplots* in the right panel (dye+) show depolarization of eleocytes (*E* *green*) and amoebocytes (*A* *blue*) (FL2-H, orange fluorescence of MiToPT^®^ TMRE vs. AF on FL1-H). Control plots of cells before addition of the dye (dye−) reveal negative signal in FL2-H (color figure online)

Mitochondrial depolarization is an important factor leading to and reflecting on, apoptotic cell death (Heiskanen et al. 1999). Similar to the results of the test for determination of cell apoptosis, we confirmed that the control animals carried vital coelomocytes as they had low ratio of depolarized mitochondrial membrane (Fig. 2a, b (dot plots); Table 1; Suppl. Fig. 2 showing representative histograms). However, the degree of depolarization was increased upon immunostimulation at any tested time point (24 or 72 h). Comparison of data for mitochondrial depolarization against the autofluorescence (AF) of eleocytes (E) revealed that the latter cells were less sensitive to the immunostimulation than amoebocytes (Fig. 2a). After 24 h of stimulation only PMA statistically significantly interfered with eleocyte mitochondrial polarization. While after 72 h, also LPS and zymosan (Z) had a similar effect to PMA (Fig. 2a). In case of amoebocytes, depolarization of mitochondria was detected in response to all stimulants but especially after PMA and zymosan (Z) treatments (Fig. 2a).

Mitochondrial depolarization is also shown on representative dot plots (the right hand side panel, dye− vs. dye+). Less cells shifted to the right upon intake of the dye in animals treated with PMA and LPS than in control animals (CTR). Only cells with polarized mitochondrial membrane revealed strong fluorescent (FL2-H) staining (Fig. 2b). Representative dot plots on the left hand side of Fig. 2b show distribution and ratio of eleocytes and amoebocytes in control animals and earthworms treated in vivo with PMA and LPS.

### Respiratory burst in coelomocytes upon immune stimulation

**Fig. 3:**
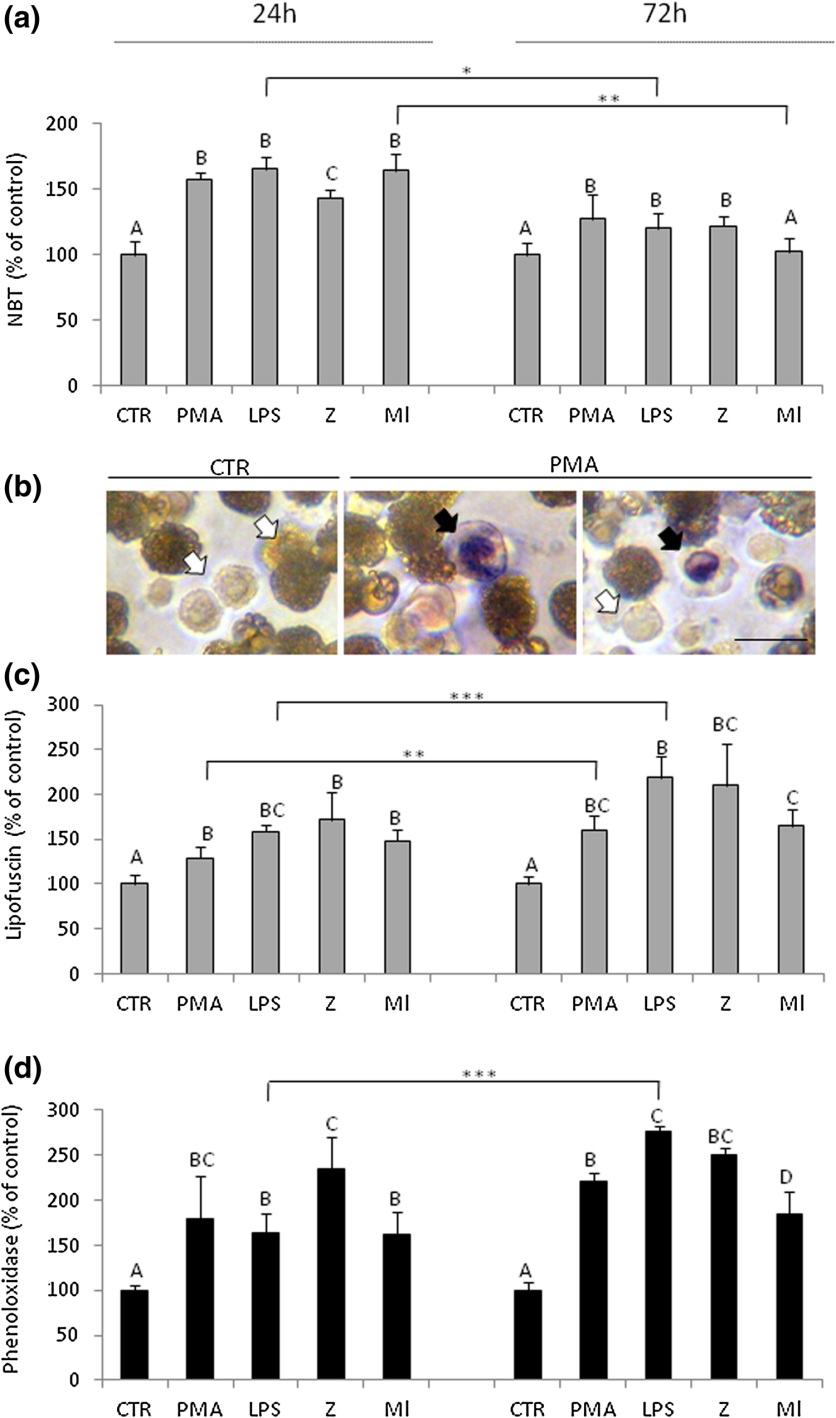
Respiratory burst, presence of lipofuscin, and phenoloxidase activity in coelomocytes upon immune stimulation. **a**–**b** Respiratory burst (NBT reduction) quantification in coelomocytes retrieved from earthworms *E. andrei* after 24 or 72 h since injection with sodium chloride (control—CTR) or PMA (0.1 µg/ml) for 24 h, LPS (1 mg/ml), zymosan (Z, 1 mg/ml) or *M. luteus* (Ml, 1 mg/ml) (**a**). Visualization (**b**) of cells undergoing the respiratory burst (*black arrows*; *dark blue* color deposit); non-active cells are marked with *white arrows*. Representative images were obtained from a control worm (CTR) and animals treated with PMA (0.1 µg/ml),* scale bar* 25 µm. **c** Presence of lipofuscin, and **d** phenoloxidase activity was assessed in coelomocytes retrieved from the same animals as in (**a**). Mean + SE, *n* = 12–16 (3–5 earthworms per experiment, each experiment was repeated 3–4 times). *Different letters* (e.g. **a** vs. **b** or *A* vs. *B*), indicate statistically significant differences between the groups at *p* < 0.05, according to one-way ANOVA, *differences statistically significant between the same groups at different time points, at *p < 0.05; **p < 0.01, ***p < 0.001, according to *t* test (color figure online)

Respiratory burst assay revealed that all used stimulants statistically significantly increased ROS generation, and this effect was stronger 24 than 72 h after the in vivo injections of LPS and *M. luteus* (Ml) (Fig. 3a). Representative images (Fig. 3b) from a control earthworm (CTR) and two animals treated with PMA show the respiratory burst reaction in individual cells. The positive reaction is especially visible in amoebocytes (black arrows; dark blue color deposit) but not in eleocytes. Respiratory burst non-active cells are marked with white arrows.

### Presence of lipofuscin and phenoloxidase activity in coelomocytes upon immune stimulation

Aware of the link between the ROS generation and accumulation and synthesis of lipofuscin (Valembois et al. 1994) we studied the latter. Our results indicate increased presence of lipofuscin in coelomocytes of immunostimulated earthworms. Lipofuscin was detected after all stimulants and its levels were higher in comparison to control animals. PMA and LPS further increased it between 24 and 72 h after the injections (Fig. 3c).

Also immune-related enzymatic activity of phenoloxidase (PO) was investigated. Presence of activity of PO in coelomocyte homogenates was detected in all samples derived from stimulated animals (Fig. 3d), but the LPS treatment further enhanced PO activity at 72 h in comparisons to 24 h.

### Activity of antioxidant enzymes: catalase (CAT) and glutathione peroxidises (GPOX and GSTPX) in coelomocytes upon immune stimulation

The intracellular concentration of ROS depends on a wide range of antioxidant systems (Abele and Puntarulo 2004; Weydert and Cullen 2010). Cells produce antioxidants to prevent, or repair, the damage caused by ROS, as well as to regulate redox-sensitive signalling pathways. The major antioxidant enzymes contained in cells that are thought to be necessary for life in all oxygen metabolizing cells are catalase (CAT) and glutathione peroxidase (GPx) (Weydert and Cullen 2010).

**Fig. 4:**
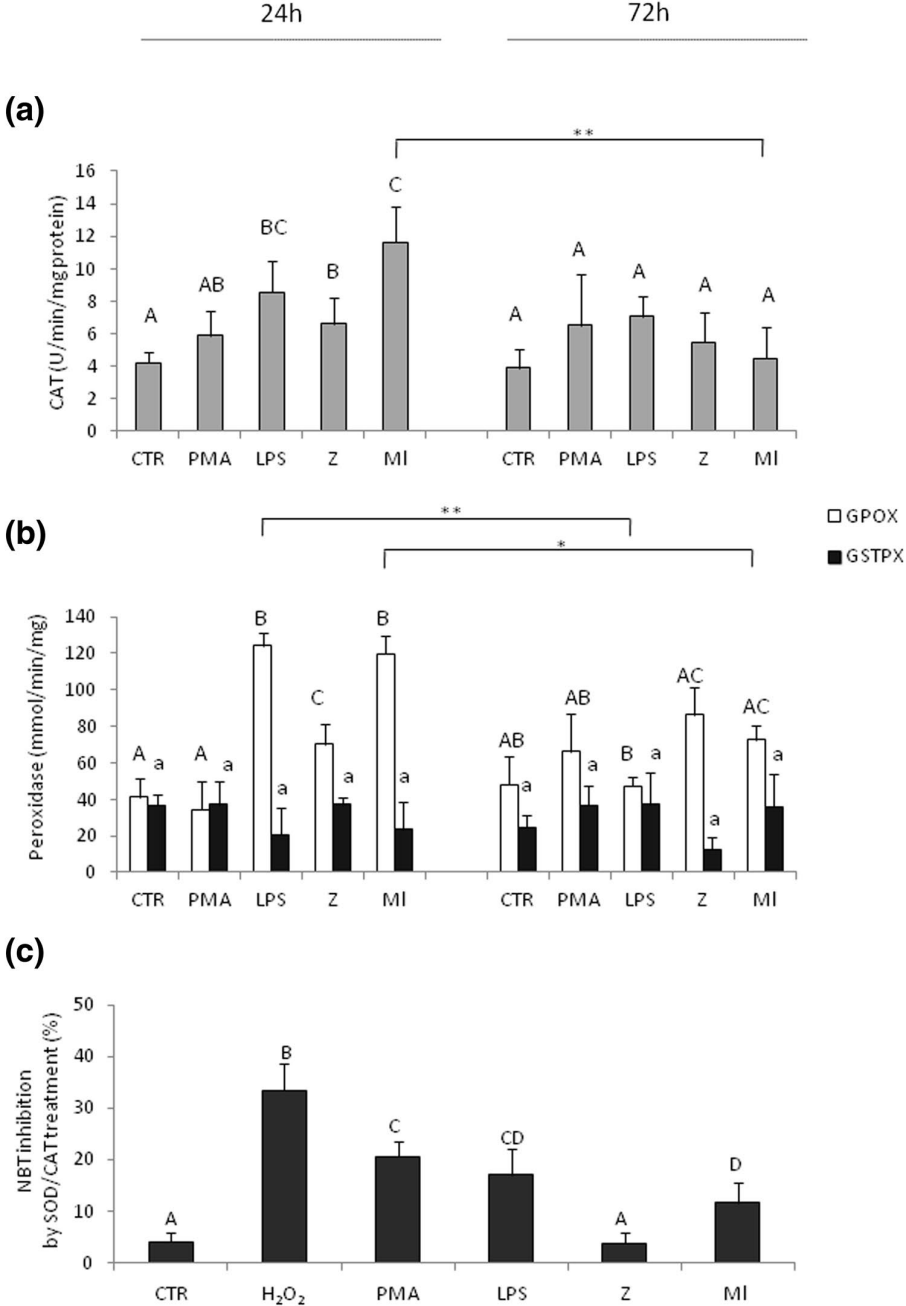
Activity of antioxidant enzymes. **a** Catalase—CAT and **b** glutathione peroxidases: selenium-independent (GSTPx) and -dependent (GPOX), in coelomocytes derived from earthworms *E. andrei* after 24 or 72 h since injection with sodium chloride (control—CTR) or PMA (0.1 µg/ml), LPS (1 mg/ml), zymosan (Z, 1 mg/ml) or *M. luteus* (Ml, 1 mg/ml). Mean + SE, *n* = 9 (three earthworms per experiment, each experiment was repeated 3 times). **c** In some studies, coelomocytes were retrieved from unstimulated *E. andrei* and studied ex vivo. The cells were preincubated for 1 h with superoxide dismutase (SOD, 3000 U) together with catalase (CAT; 3000 U), and subsequently stimulated with H_2_O_2_ (100 mMol, as positive control), PMA (10 µg/ml), LPS (1 mg/ml), Z (1 mg/ml) or Ml (1 mg/ml). Then, NBT test was performed to evaluate effects of the SOD/CAT pretreatment on the respiratory burst. Data are expressed as percentage of respiratory burst inhibition by the antioxidants. Mean + SE, *n* = 6 (two earthworms per experiment, each experiment was repeated 3 times). *Different letters* (e.g. **a** vs. **b** or *A* vs. *B*), indicate statistically significant differences between the groups at *p* < 0.05, according to one-way ANOVA, *differences statistically significant between the same groups at different time points, at *p < 0.05; **p < 0.01, according to *t* test

Catalase activity was increased only 24 h post immunostimulants, and after application of LPS, zymosan (Z), *M. luteus* (Ml) but not PMA. The highest CAT activity, over two fold greater than in the control cells, was determined in the samples collected from animals injected with LPS and Ml for 24 h (Fig. 4a). Furthermore, selenium depended glutathione peroxidase (GPOX), but not selenium independent (GSTPX), have proved to be up-regulated by the immunostimulants (Fig. 4b). Moreover, the data showed highly positive correlation between catalase (CAT) activity, and selenium dependent glutathione peroxidase (GPOX), as it was especially increased upon LPS and *M. luteus* (Ml) after 24 h of in vivo stimulation (Fig. 4a vs. b; Table 1). Additional, zymosan (Z), but not PMA, enhanced GPOX activity (Fig. 4b).

In an additional experimental setting, coelomocytes obtained from unstimulated *E. andrei* were studied ex vivo. They were preincubated for 1 h with superoxide dismutase (SOD, 3000 U) together with catalase (CAT) and subsequently stimulated with H_2_O_2_ (100 mM, as positive control), PMA, LPS, zymosan (Z) or *M. luteus* (Ml). After stimulation, NBT test was performed to evaluate effects of the SOD/CAT pretreatment. We detected that ex vivo incubation with immunostimulants increased statistically significantly respiratory burst, but pretreatment of cells with the free-radical scavenger SOD/CAT partially inhibited this process in a stimulant dependent manner, in order of strongest inhibition to lowest: Z < Ml < LPS < PMA < H_2_O_2_ (Fig. 4c).

### Effects of secondary immunostimulation on respiratory burst in coelomocytes

**Fig. 5:**
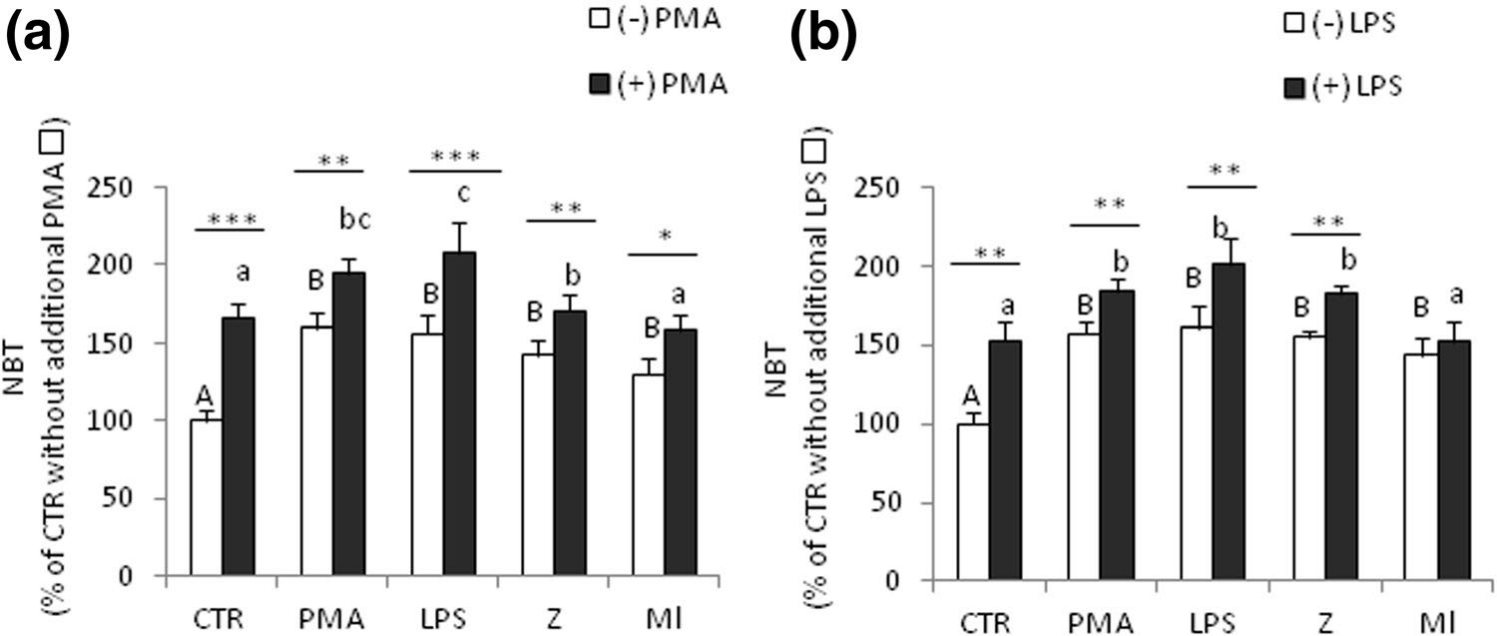
Effects of secondary immunostimulation on respiratory burst in coelomocytes. Earthworms were stimulated in vivo for 24 h with either sodium chloride (control—CTR) or PMA (0.1 µg/ml), LPS (1 mg/ml), zymosan (Z, 1 mg/ml) or *M. luteus* (Ml, 1 mg/ml). Subsequently, coelomocytes were isolated and further studied ex vivo. Some cells were then additionally stimulated for 1 h with PMA (10 µg/ml) or LPS (10 µg/ml) [(+) PMA, (+) LPS, respectively] or with adequate control media [(−) PMA, (−) LPS, respectively]. Mean + SE, *n* = 12–16 (3–5 earthworms per experiment, each experiment was repeated 3–4 times). *Different letters* (e.g. **a** vs. **b** or *A* vs. *B*) indicate statistically significant differences between the groups at *p* < 0.05, according to one-way ANOVA, *differences statistically significant between groups with identical primary stimulation but with (+) or without (−) additional stimulation, at *p < 0.05; **p < 0.01; ***p < 0.001, according to *t* test

The priming action of in vivo applied stimulants was tested further by incubation of coelomocytes ex vivo with secondary stimuli, PMA or LPS. At first, the earthworms were immunostimulated in vivo with sodium chloride (control—CTR) or PMA, LPS, zymosan (Z) or *M. luteus* (Ml) for 24 h, then their coelomocytes were isolated and stimulated ex vivo for 1 h with PMA or LPS. The strongest effect of a second stimulation on the respiratory burst was detected in the case of cells that were primarily stimulated with LPS and PMA (Fig. 5a, b). And coelomocytes collected from LPS injected earthworms were the most active in the production of ROS, regardless of the nature of the second stimulant. Coelomocytes collected from animals injected primarily with *M. luteus* (Ml) were generally insensitive to the secondary stimulation (Fig. 5a, b).

## Discussion

Acute, rapid but shortly resolved, immune response is a favorable response of the body securing survival of the affected host. It is meant to serve two purposes, eliminate the cause of the immune response (invading pathogen or mechanistic insult) and heal the wounds when infection/insult is eliminated. The former of the actions can, however, lead to bystander damage of the host cells (Bresnahan and Tanumihardjo 2014). It can be due to the pathogen itself (e.g. its toxins) but foremost due to the products generated during the immune response. These can be cytokines, eicosanoids, neutrophil extracellular traps (NETs) and especially, reactive oxygen species (ROS) generated during the respiratory burst (Fialkow et al. 2007; Kolaczkowska et al. 2015). The latter process seems to be very effective in pathogen elimination but it is strongly associated with some of the most harmful side effects of immune reaction, and is common for both vertebrates and invertebrates (Holmström and Finkel 2014). Therefore to minimize the “one must pay the price” effect of ROS generation, evolution favored development of antioxidative systems (Holmström and Finkel 2014).

Here we asked how efficient is the antioxidant response to the generation of ROS in earthworms and if their release can indeed prevent or minimize damage to coelomocytes which generate them. We conducted our studies on epigeic species *Eisenia andrei* which can be found in a wide range of habitats, feeds on, and thus stays in constant contact, with soil which is rich in microbes (Sizmur et al. 2011). Moreover, we and others have shown previously that the species is sensitive to multiple immune (Homa et al. 2013) and environmental factors (Gambi et al. 2007; Velki and Hackenberger 2013). In the current study, we used both physiological and non-physiological, but potent, stimulants of the respiratory burst to investigate mechanisms of their action in terms of the antioxidative response. As the latter agent, we used synthetic phorbol-12-myristate-13-acetate (PMA) known to strongly induce ROS in vertebrate cells (Kepka et al. 2014) but also in earthworm coelomocytes (Opper et al. 2010; Homa et al. 2013). Having PMA as a reference we subsequently focused on natural pathogen-derived ROS inducers, lipopolisaccharide (LPS) of Gram^−^-origin, Gram+ *Micrococcus luteus* and zymosan, glucan derived from fungi/yeast (*S. cerevisiae*) cell wall (‎Kolaczkowska 2002; Jiang et al. 2013; Tokura et al. 2014). The stimulants were injected into the coelom of the earthworms to mimic in vivo situation when pathogens enter the body in a more dramatic way than through dermal exposure (e.g. with digested soil or through open wounds). We observed that all factors induced similarly potent respiratory burst that was not silenced even 3 days after the insult. This strongly correlated with significantly lowered numbers of coelomocytes that were retrieved from the coelomic fluid. This effect can be correlated to ROS production as the oxidants not only eliminate pathogens but also activate signaling pathways which lead to cell death and/or stimulate cell proliferation (Kang et al. 2015). For example, a similar correlation was shown in *Eisenia hortensis* in which heat stress-induced ROS production lead to decreased coelomocyte viability (Tumminello and Fuller-Espie 2013).

Not surprisingly, the cells that survived the contact with the stimulant were less viable than in unstimulated controls, and many of them entered the apoptotic pathway. Our studies which evaluated mitochondrial membrane polarization further clarified that most of the cells that entered the apoptotic pathway were amoebocytes, with eleocytes being more resistant to the immune stimulation. Such polarization was observed previously by Nacarelli and Fuller-Espie (2011) in coelomocytes from *Eisenia hortensis* incubated with zymosan. Although the coelomocyte polarization is clearly existent, it might differ between species and/or insults as just recently eleocytes of *E. fetida* were shown to be more sensitive to metal exposure (Irizar et al. 2015).

Interestingly, we did not observe significant changes in numbers of cells dying simply by necrosis. This is indeed worth noticing as the necrotic death, unlike apoptosis, is secondarily activating phagocytes due to the exposure of damage-associated molecular pattern molecules or DAMPs (Kono and Rock 2008). This indicates that coelomocytes of *E. andrei* are rather prone to the programmed cell death than simple disintegration upon contact with bacterial and fungal products. The lower numbers of coelomocytes that we retrieved from the stimulated animals could be due to their prior death, but the cells might have also been expelled through pores in the integument which is a common response of the earthworm to the insult of any kind (Sims, and Gerard 1985). Another possibility is that some of the cells formed brown bodies i.e. coelomocyte aggregates encapsulating pathogens, and thus are not detectable as single cells (Valembois et al. 1994; Cooper 1996). Although both cell types are involved in formation of brown bodies, eleocytes are more noticeable (Cooper 1996). In fact, we observed more aggregates (early brown bodies) of coelomocytes collected from earthworms stimulated with LPS, Z and Ml than with PMA but especially than control animals (unpublished observation).

The correlation “insult—less (viable) cells—more cells entering apoptosis” was not however so clearly observed upon injection of PMA in long term. Actually we observed that although after first 24 h numbers of amoebocytes indeed decreased in earthworms injected with PMA, 2 days later they were increased despite a fact that numerous cells were still less viable and apoptotic. We propose that this could be due to the PMA capacity to upregulate coelomocyte proliferation. Previously we studied effects of dermal exposure of *E. andrei* to some immune stimulants, including PMA and LPS, and we detected that the former compound induced much stronger proliferation of the cells after 3 days (Homa et al. 2013). Moreover, the division of amoebocytes dominated over eleocytes. Although these studies cannot be simple extrapolated to the current experimental setting, they do indicate the proliferative potential of PMA, especially towards amoebocytes. In fact, PMA was shown previously to be highly mitogenic e.g. in birds (McNeilly et al. 1999). And vast regenerative capacity of earthworms to rebuild the pool of coelomocytes is well recognized (Homa et al. 2008). Another observation unique to PMA stimulated earthworms is that although ROS generation was at the similar level as upon other factors, the animals lost more cells and had highest numbers of apoptotic coelomocytes within first 24 h. We will discuss this observation in the light of other findings later.

The process of melanization is key to the defense against a wide range of pathogens and results in deposition of melanin on the microbe (Valembois et al. 1994). Also lipofuscin is deposited in brown bodies and a moderate production of ROS was also shown to occur when eleocytes aggregate together (Valembois and Lassègues 1995). In the current study we did observe its increased deposition, and lipofuscin was further accumulating in time which corresponds to increasing formation of brown bodies. Process of melanization is controlled by the prophenoloxidase system (pro-PO) which can be compared with complement system of vertebrates (Smith 1996). Although unlike in insects, PO activation in earthworms takes more time and is weaker, it still plays a significant role (Procházková et al. 2006). Again, increasing activity of PO was observed upon all tested immune stimulants, but it was strongest after LPS and zymosan injections. This can be explained by a fact that coelomic cytolytic factor (CCF-1) of earthworms recognizing foreign particles and initiating pro-PO activation particularly efficiently binds both β-1,3-glucan (present in zymosan) and LPS (Beschin et al. 1998; Kolaczkowska 2002).

Finally we focused on generation of antioxidants and their impact on the ROS generation and fate of coelomocytes. Our investigations were inspired by a fact that very few studies on antioxidant enzymes in annelids have been performed up till now. The antioxidant enzymes neutralize superoxide and hydrogen peroxide which are scavenged by superoxide dismutases (SOD) and catalyze (CAT), respectively. The former enzyme exists in earthworms in two forms, selen-independent (GPOX) and -dependent (GSTPX) (Saint-Denis et al. 1998). Our studies revealed that of the two, only GPOX activity was increased upon stimulation with LPS, zymosan and *M. luteus*. This correlates with an earlier report on a strong selenium-independent peroxidase activity in coelomic fluid of *E.f. andrei* (Milochau et al. 1997). Also catalase activity was confirmed to operate in this species and possess unique double characteristics of catalytic and peroxidative activities (Saint-Denis et al. 1998). In line with this, we observed enhanced CAT activity within the first 24 h post stimulation. Importantly, we did not detect either GPOX or CAT activity in coelomocytes retrieved from animals injected with PMA. Therefore although ROS generation was profound upon this insult, the antioxidative system was either not initiated or promptly switched off. In fact, the enzymes might be deactivated by some strong stimuli, e.g. heavy metals (Labrot et al. 1996). Another possibility is that coelomocytes exposed to certain agents are insensitive to antioxidants. To test this hypothesis we retrieved coelomocytes from earthworms stimulated in vivo with each of the compounds, treated them ex vivo with a mixture of SOD and CAT, and tested their ability to produce ROS. Although indeed coelomocytes showed different sensitivity to the antioxidants depending on the respiratory burst stimulant (Z > *Ml* > PMA = LPS), PMA-primed cells were equally sensitive to SOD/CAT as those treated with LPS. These data suggest that PMA does not trigger antioxidant production rather than causes cell insensitivity towards them. However, independently of the cause of such effect, we saw a clear inverse correlation between inactive antioxidant system of PMA-stimulated coelomocytes and their stronger decline and entering of the apoptotic pathway. Clearly, the lack of action of CAT and GPOX impacted the fate of the cells, indicating importance of the system in providing protection for the immunocompetent cells of the earthworms.

Living in soil, earthworms are in constant contact with diverse pathogens and one cannot exclude a possibility that they are infected by different pathogens simultaneously or shortly one after another. For this we also tested how repeated exposure to similar or different immune stimulation will affect the capacity of coelomocytes to perform consecutive respiratory burst. It turned out that although primarily-unstimulated cells then secondarily-stimulated with either LPS or PMA revealed strong ROS production, almost all tested combinations (e.g. LPS–LPS, LPS–Z, Ml–PMA) still induced a strong second hit of the respiratory burst. The strongest capacity was revealed for cells initially stimulated with LPS, and weakest for *M. luteus*. While LPS is one of the strongest immune stimulants known (e.g. Wittwer et al. 1997), *M. luteus* can release its own catalyze to neutralize ROS (Marie and Parak 1980).

In summary, here we confirmed activation of reactive oxygen species generation in response to pathogens or their derivatives to which the earthworms are exposed in soil. The ROS generation correlated with lipofuscin and prophenoloxidase activities as all of them participate in elimination of the insult. Importantly, the compounds simultaneously activated the antioxidant system which could not completely prevent, yet significantly diminished, the damage to coelomocytes and their death. This notion is strengthened by observation that when the antioxidative system is not initiated (PMA), much more coelomocytes dies. We have also showed that the coelomocyte capacity to produce ROS in short intervals, and in response to differential agents, is not compromised in *E. andrei* by the first activation of the respiratory burst. The lack of exhausted phenotype confirms that coelomocytes are prepared and armed to be constantly at bay to the next possible lethal attack.

Overall, the current study reveals evolutionary stress for concomitant development of the oxidative and antioxidative systems which only together provide efficient self-protection of the body, independently of the organism complexity.

## Electronic supplementary material

Below is the link to the electronic supplementary material.

Supplementary material 1 (TIFF 147 kb)

Supplementary material 2 (TIFF 181 kb)

## References

[CR1] Abele D, Puntarulo S (2004). Formation of reactive species and induction of antioxidant defence systems in polar and temperate marine invertebrates and fish. Comp Biochem Physiol A Mol Integr Physiol.

[CR2] Beschin A, Bilej M, Hanssens F, Raymakers J, Van Dyck E, Revets H, Brys L, Gomez J, De Baetselier P, Timmermans M (1998). Identification and cloning of a glucan- and lipopolisacharyde-binding protein from *Eisenia foetida* earthworm involved in the activation of prophenoloxidase cascade. J Biol Chem.

[CR3] Bilej M, De Baetselier P, Beschin A (2000). Antimicrobal defense of the earthworm. Folia Microbiol.

[CR4] Bilej M, De Baetselier P, Van Dijck E, Stijlemans B, Colige A, Beschin A (2001). Distinct carbohydrate recognition domains of an invertebrate defense molecule recognize Gram-negative and Gram-positive bacteria. J Biol Chem.

[CR5] Bradford MM (1976). Rapid and sensitive method for the quantitation of microgram quantities of protein utilizing the principle of protein-dye binding. Anal Biochem.

[CR6] Bresnahan KA, Tanumihardjo SA (2014). Undernutrition, the acute phase response to infection, and its effects on micronutrient status indicators. Adv Nutr.

[CR8] Chadzinska M, Savelkoul HF, Verburg-van Kemenade BML (2009). Morphine affects the inflammatory response in carp by impairment of leukocyte migration. Dev Comp Immunol.

[CR9] Cooper EL, Rencevich B, Muller WEG (1996). Earthworm immunology. Invertebrate immunology.

[CR11] Fialkow L, Wang Y, Downey GP (2007). Reactive oxygen and nitrogen species as signaling molecules regulating neutrophil function. Free Radic Biol Med.

[CR12] Fugère N, Brousseau P, Krzystyniak K, Coderre D, Fournier M (1996). Heavy metal-specific inhibition of phagocytosis and different in vitro sensitivity of heterogeneous coelomocytes from *Lumbricus terrestris* (Oligochaeta). Toxicol.

[CR13] Gambi N, Pasteris A, Fabbri E (2007). Acetylcholinesterase activity in the earthworm (*Eisenia andrei*) at different conditions of carbaryl exposure. Comp Biochem Physiol C.

[CR14] Green DR, Reed JC (1998). Mitochondria and apoptosis. Science.

[CR15] Haugland GT, Jakobsen RA, Vestvik N, Ulven K, Stokka L, Wergeland HI (2012). Phagocytosis and respiratory burst activity in lumpsucker (*Cyclopterus lumpus* L.) leucocytes analysed by flow cytometry. PLoS One.

[CR16] Heiskanen KM, Bhat MB, Wang HW, Ma J, Nieminen AL (1999). Mitochondrial depolarization accompanies cytochrome c release during apoptosis in PC6 cells. J Biol Chem.

[CR17] Holmström KM, Finkel T (2014). Cellular mechanisms and physiological consequences of redox-dependent signalling. Nat Rev Mol Cell Biol.

[CR18] Homa J, Bzowska M, Klimek M, Plytycz B (2008). Flow cytometric quantification of proliferating coelomocytes non-invasively retrieved from the earthworm, *Dendrobaena veneta*. Dev Comp Immunol.

[CR19] Homa J, Stürzenbaum SR, Morgan AJ, Plytycz B (2007). Disrupted homeostasis in coelomocytes of *Eisenia fetida* and *Allolobophora chlorotica* exposed dermally to heavy metals. Europ J Soil Biol.

[CR20] Homa J, Zorska A, Wesolowski D, Chadzinska M (2013). Dermal exposure to immunostimulants induces changes in activity and proliferation of coelomocytes of *Eisenia andrei*. J Comp Physiol B.

[CR21] Irizar A, Rivas C, García-Velasco N, de Cerio FG, Etxebarria J, Marigómez I, Soto M (2015). Establishment of toxicity thresholds in subpopulations of coelomocytes (amoebocytes vs. eleocytes) of *Eisenia fetida* exposed in vitro to a variety of metals: implications for biomarker measurements. Ecotoxicology.

[CR22] Jandova J, Janda J, Sligh JE (2013). Cyclophilin 40 alters UVA-induced apoptosis and mitochondrial ROS generation in keratinocytes. Exp Cell Res.

[CR23] Jiang Q, Zhou Z, Wang L, Shi X, Wang J, Yue F, Yi Q, Yang C, Song L (2013). The immunomodulation of inducible nitric oxide in scallop *Chlamys farreri*. Fish Shellfish Immunol.

[CR24] Kang S, Han J, Song SY, Kim WS, Shin S, Kim JH, Ahn H, Jeong JH, Hwang SJ, Sung JH (2015). Lysophosphatidic acid increases the proliferation and migration of adipose derived stem cells via the generation of reactive oxygen species. Mol Med Rep.

[CR25] Katz ML, Robinson WG (2002). What is lipofuscin? Defining characteristics and differentation from other autofluorescent lysosomal storage bodies. Arch Gerontol Geriat.

[CR26] Kauschke E, Mohrig W, Cooper EL (2007). Coelomic fluid proteins as basic components of innate immunity in earthworms. Eur J Soil Biol.

[CR27] Kepka M, Verburg-van Kemenade BM, Homa J, Chadzinska M (2014). Mechanisms involved in apoptosis of carp leukocytes upon in vitro and in vivo immunostimulation. Fish Shellfish Immunol.

[CR28] Kettle AJ, Winterbourn CC (1997). Myeloperoxidase: a key regulator of neutrophil oxidant production. Redox Rep.

[CR29] Kolaczkowska E (2002). Shedding light on vascular permeability during peritonitis: role of mast cell histamine versus macrophage cysteinyl leukotrienes. Inflamm Res.

[CR30] Kolaczkowska E, Jenne CN, Surewaard BG, Thanabalasuriar A, Lee WY, Sanz MJ, Mowen K, Opdenakker G, Kubes P (2015). Molecular mechanisms of NET formation and degradation revealed by intravital imaging in the liver vasculature. Nat Commu.

[CR31] Kono H, Rock KL (2008). How dying cells alert the immune system to danger. Nat Rev Immunol.

[CR32] Kurek A, Homa J, Kauschke E, Plytycz B (2007). Characteristics of coelomocytes of the stubby earthworm, *Allolobophora chlorotica* (Sav.). Eur J Soil Biol.

[CR33] Labrot F, Ribera D, Saint-Denis M, Narbonne JF (1996). *In vitro* and in vivo studies of potential biomarkers of lead and uranium contamination: lipid peroxidation, acetylcholinesterase, catalase and glutathione peroxidase activities in three non-mammalian species. Biomarkers.

[CR34] Marie AL, Parak F (1980). Factors affecting the growth and the catalase synthesis in *Micrococcus luteus* cells. Hoppe-Seyler’s Zeitschrift fur physiologische Chemie.

[CR35] McNeilly F, Walker I, Allan GM, Adair BM (1999). Bursal lymphocyte proliferation in the presence of phorbol myristate acetate: effect of IBDV strains on the proliferation response. Avian Pathol.

[CR36] Milochau A, Lasségues M, Valembois P (1997). Purification, characterization and activities of two hemolytic and antibacterial proteins from coelomic fluid of the annelid *Eisenia fetida andrei*. Biochim Biophys Acta.

[CR37] Moore MN (1988). Cytochemical responses of the lysosomal system and NADPH-ferrihemoprotein reductase in molluscan digestive cells to environmental and experimental exposure to xenobiotics. Mar Ecol Prog Ser.

[CR38] Nacarelli T, Fuller-Espie SL (2011). Pathogen-associated molecular pattern-induced mitochondrial membrane depolarization in the earthworm *Eisenia hortensis*. J Invertebr Pathol.

[CR39] Opper B, Németh P, Engelmann P (2010). Calcium is required for coelomocyte activation in earthworms. Mol Immunol.

[CR40] Pothi R (2013). Anti-oxidant enzyme levels and quantification of reactive oxygen species in *Mycobacterium aurum*. IJAR.

[CR41] Pragya P, Shukla AK, Murthy RC, Abdin MZ, Chowdhuri DK (2014). Over-expression of superoxide dismutase ameliorates Cr(VI) induced adverse effects via modulating cellular immune system of *Drosophila melanogaster*. PLoS One.

[CR42] Procházková P, Šilerová M, Stijlemans B, Dieu M, Halada P, Josková R, Beschin A, Baetselier De, Bilej M (2006). Evidence for proteins involved in prophenoloxidase cascade *Eisenia fetida* earthworms. J Comp Physiol B.

[CR43] Saint-Denis M, Labrot F, Narbonne JF, Ribera D (1998). Glutathione, glutathione-related enzymes, and catalase activities in the earthworm *Eisenia fetida andrei*. Arch Environ Contam Toxicol.

[CR44] Salganik RI (2001). The benefits and hazards of antioxidants: controlling apoptosis and other protective mechanisms in cancer patients and the human population. J Am Coll Nutr.

[CR45] Sigfrid LA, Cunningham JM, Beeharry N, Lortz S, Tiedge M, Lenzen S, Carlsson C, Green JC (2003). Cytokines and nitric oxide inhibit the enzyme activity of catalase but not its protein or mRNA expression in insulin-producing cells. J Mol Endocrinol.

[CR47] Sims RW, Gerard BM (1985) Earthworms: keys and notes for the identification and study of the species. In: DM Kermack, RSK Barnes (eds) Synopsis o f British fauna (new series), vol 31. E.J. Brill/Dr. W. Backhuys, London, pp 6

[CR48] Sizmur T, Tilston EL, Charnock J, Palumbo-Roe B, Watts MJ, Hodson ME (2011). Impacts of epigeic, anecic and endogeic earthworms on metal and metalloid mobility and availability. J Environ Monit.

[CR49] Smith VJ (1996). The prophenoloxidase activating system: a common defence pathway for deuterostomes and protostomes?. Adv Comp Environ Physiol.

[CR50] Söderhäll K (eds) (2010) Invertebrate Immunity. Advances in Experimental Medicine and Biology. Springer US. 708, 1-316 21528689

[CR51] Terman A, Brunk UT (2004). Lipofuscin. Int J Biochem Cell Biol.

[CR52] Tokura A, Fu GS, Sakamoto M, Endo H, Tanaka S, Kikuta S, Tabunoki H, Sato R (2014). Factors functioning in nodule melanization of insects and their mechanisms of accumulation in nodules. J Insect Physiol.

[CR53] Tumminello RA, Fuller-Espie SL (2013). Heat stress induces ROS production and histone phosphorylation in celomocytes of *Eisenia hortensis*. ISJ.

[CR54] Valembois P, Lassègues M (1995). *In vitro* generation of reactive oxygen species by free coelomic cells of the annelid *Eisenia fetida andrei*: an analysis by chemiluminescence and nitro blue tetrazolium reduction. Dev Comp Immunol.

[CR55] Valembois P, Lassègues M, Roch P (1992). Formation of brown bodies in the coelomic cavity of the earthworm *Eisenia fetida andrei* and attendant changes in shape and adhesive capacity of constitutive cells. Dev Comp Immunol.

[CR56] Valembois P, Seymour J, Lasségues M (1994). Evidence of lipofuscin and melanin in the brown body of the earthworm *Eisenia fetida andrei*. Cell Tissue Res.

[CR57] Vargas-Albores F, Yepiz-Plascencia G (2000). Beta glucan binding protein and its role in shrimp immune response. Aquaculture.

[CR58] Velki M, Hackenberger BK (2013). Different sensitivities of biomarker responses in two epigeic earthworm species after exposure to pyrethroid and organophosphate insecticides. Arch Environ Contam Toxicol.

[CR59] Weydert CJ, Cullen JJ (2010). Measurement of superoxide dismutase, catalase and glutathione peroxidase in cultured cells and tissue. Nat Protoc.

[CR60] Wilczek G, Babczyńska A, Wilczek P (2013). Antioxidative responses in females and males of the spider *Xerolycosa nemoralis* (Lycosidae) exposed to natural and anthropogenic stressors. Comp Biochem Physiol C Toxicol Pharmacol.

[CR61] Wittwer D, Weise C, Götz P, Wiesner A (1997). LPS (lipopolysaccharide)-activated immune responses in a hemocyte cell line from *Estigmene acraea* (Lepidoptera). Dev Comp Immunol.

[CR62] Zimmermann M, Meyer N (2011). Annexin V/7-AAD staining in keratinocytes. Methods Mol Biol.

