# Supplementary figures and images for: Effective activation of antioxidant system by immune-relevant factors reversely correlates with apoptosis of Eisenia andrei coelomocytes
Source: J Comp Physiol B. 2016 Feb 27;186:417–30. doi: 10.1007/s00360-016-0973-5 (PMC4830880; doi:10.1007/s00360-016-0973-5)

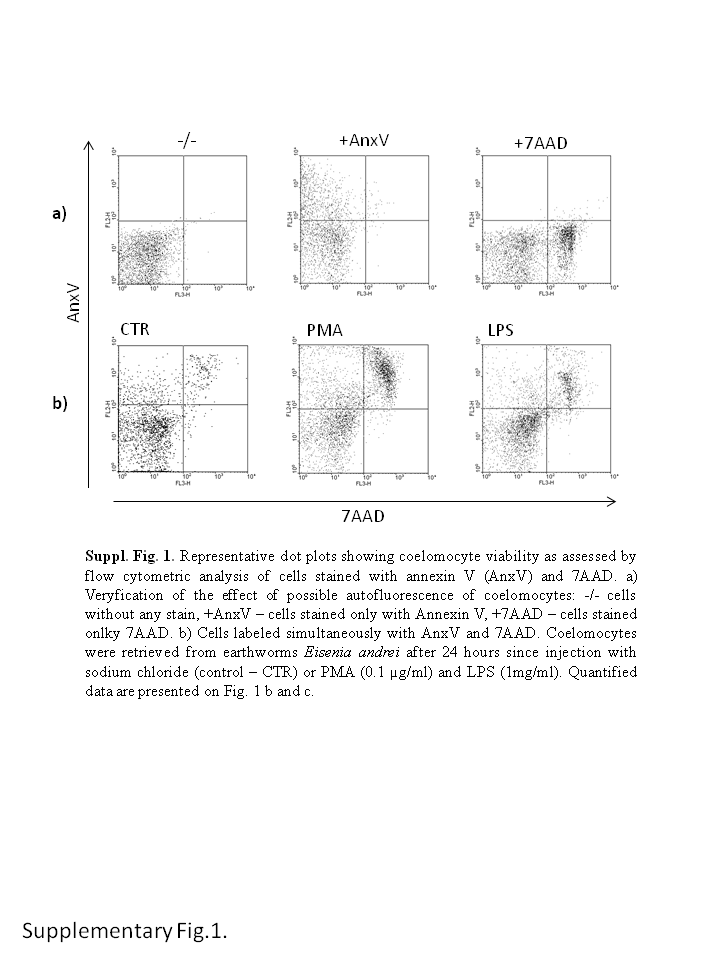

Supplement: Supplementary file 1 — Supplementary material 1 (TIFF 147 kb) [file 360_2016_973_MOESM1_ESM.tif]

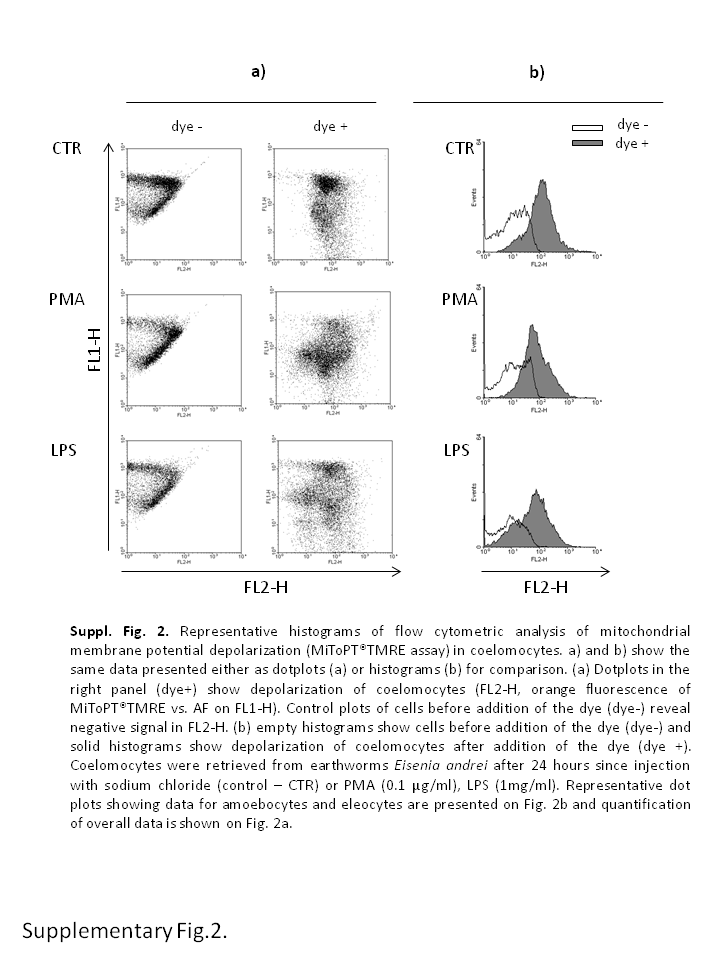

Supplement: Supplementary file 2 — Supplementary material 2 (TIFF 181 kb) [file 360_2016_973_MOESM2_ESM.tif]
